# Integrated Microbiological, Physicochemical, and Sensory Assessment of Shrimp Quality During Commercial Iced Storage: Implications for Shelf-Life Evaluation and Freshness Indicators

**DOI:** 10.3390/microorganisms14061266

**Published:** 2026-06-04

**Authors:** Michela Pellegrini, Debbie Andyanto, Asia Petozzi, Lucilla Iacumin, Cristian Edoardo Maria Bernardi, Giuseppe Comi

**Affiliations:** 1 Department of Agricultural, Food, Environmental and Animal Sciences, University of Udine, Via Sondrio 2/a, 33100 Udine, Italy; debbie.andyanto@uniud.it (D.A.); petozzi.asia@spes.uniud.it (A.P.); lucilla.iacumin@uniud.it (L.I.); 2 Department of Veterinary Medicine and Animal Sciences, University of Milan, Via dell’Università 6, 26900 Lodi, Italy; cristian.bernardi@unimi.it

**Keywords:** *Parapenaeus longirostris*, *Melicertus kerathurus*, cold storage, TVB-N, formaldehyde, formic acid, TBARS, microbial load

## Abstract

Shrimp are among the most valuable seafood commodities worldwide, but are also highly perishable, making their quality preservation a critical issue for both food safety and supply chain sustainability. The rapid deterioration of fresh shrimp contributes to significant post-harvest losses, highlighting the need for reliable freshness indicators capable of supporting shelf-life assessment under commercial conditions. This study evaluated the evolution of microbiological, physicochemical, and sensory parameters in two commercially important Mediterranean shrimp species, *Parapenaeus longirostris* and *Melicertus kerathurus*, stored on ice for up to 15 days under retail-like conditions. Microbial load, pH, total volatile basic nitrogen (TVB-N), thiobarbituric acid reactive substances (TBARS), formaldehyde, formic acid, and sensory attributes were monitored during storage. Microbial populations increased progressively over time but remained below commonly accepted spoilage thresholds, while physicochemical indicators showed significant changes associated with post-mortem biochemical processes. In particular, TVB-N, pH, and formic acid increased during storage, whereas formaldehyde levels decreased, suggesting the progressive transformation of trimethylamine-N-oxide degradation products. Sensory analysis indicated that shrimp maintained high quality up to approximately 12 days of iced storage, whereas samples stored for 15 days approached the limit of consumer acceptability. The combined behaviour of microbial, chemical, and sensory indicators highlights the importance of a multidimensional approach for evaluating shrimp freshness under commercial storage conditions. Based on the experimental dataset, practical reference ranges for key quality parameters are proposed (pH < 7.4; TVB-N ≤ 30 mg N/100 g; formaldehyde < 10 mg/kg; formic acid < 18 mg/kg). These results may support improved freshness evaluation, contribute to more accurate shelf-life estimation, and help reduce unnecessary seafood waste within the supply chain.

## 1. Introduction

Shrimp are aquatic crustaceans belonging to the order Decapoda and represent one of the most important seafood commodities in global fisheries and aquaculture production. Among penaeid shrimps, *Parapenaeus longirostris* (deep-water rose shrimp) and *Melicertus kerathurus* (caramote prawn) are two species of major commercial relevance in Mediterranean markets, including Italy, where they are widely sold either fresh on ice or frozen [1,2,3,4]. In recent decades, shrimp production has increased considerably due to the expansion of capture fisheries and aquaculture activities, reflecting the growing consumer demand for seafood products characterized by high nutritional value and desirable sensory properties [5,6]. Shrimps are particularly appreciated by consumers because of their high protein content, low lipid levels, and significant amounts of essential nutrients, including polyunsaturated fatty acids, vitamins, and trace elements such as zinc, iron, and selenium, and they are preferred by consumers due to their low caloric and fat content [5,6,7]. *P. longirostris* (Lucas, 1846) is listed as a priority species by the General Fisheries Commission for the Mediterranean (GFCM), and its total landings in the Mediterranean increased from approximately 7000 tons in 1970 to 22,000 tons in 2021 [3]. *M. kerathurus* (Forskål, 1775), the striped or caramote prawn, is a large penaeid shrimp inhabiting coastal marine and brackish waters of the eastern Atlantic and the entire Mediterranean Sea. Global shrimp production—comprising both capture fisheries and aquaculture—has grown substantially over recent decades, driven primarily by the expansion of intensive aquaculture in Asia and Latin America. Farmed shrimp production alone reached approximately 1.91 million tons in 2025 [3]. Global shrimp imports total 3.74 million tons in 2024, a decline of 1.6% in volume and 5.9% in value (USD 25.4 billion) compared to 2023 [3]. The leading importing markets by value were the United States (USD 6.33 billion), China (USD 5.06 billion), and Japan (USD 1.92 billion) [3]. In Asia, shrimp consumption is particularly high in China, Japan, South Korea, Vietnam, and Southeast Asian countries. In Europe, consumption is concentrated in Spain, France, Italy, Denmark, and the Netherlands. In North America, shrimp remains the most consumed seafood species in the United States. Within the Mediterranean context, wild-caught penaeid shrimp, including *P. longirostris* and *M. kerathurus*, contribute significantly to regional seafood consumption. Mediterranean landings of *P. longirostris* reached approximately 22,000 tons in 2021, up from 7000 tons in 1970, reflecting both increased fishing effort and expansion of trawl fisheries across the basin [4]. In the Mediterranean region, wild-caught penaeid shrimps contribute significantly to regional seafood consumption and represent an economically valuable resource for coastal fisheries. For example, *Parapenaeus longirostris* has been identified as a priority species within Mediterranean fisheries management frameworks, and its annual landings have increased markedly over the past decades [3,4,8]. These species are commonly harvested by trawl fisheries and distributed through relatively short supply chains, where they are typically transported in polystyrene boxes containing ice and subsequently marketed fresh in retail seafood departments. However, as consumer demand persists throughout the year, they are also frequently frozen. Specifically, in specialized Italian processing facilities, 1 day after harvest, the shrimps were singularly and rapidly treated by IQF (individual quick freezing). Shrimp are placed on conveyor belts, where a flow of very cold air (−30/−40 °C) circulates at high speed. Then they are packaged in polyethylene terephthalate (PET) film and stored at −18 °C until cooking.

Under these commercial conditions, the maintenance of adequate temperature and hygienic practices is essential to preserve shrimp quality until consumption.

Despite their nutritional and commercial importance, shrimps are among the most perishable seafood products. Their high-water activity, neutral pH, and abundance of water-soluble non-protein nitrogen compounds, including trimethylamine-N-oxide (TMAO), adenosine triphosphate (ATP), and free amino acids, favour rapid microbial growth and enzymatic degradation immediately after harvest [9,10,11,12]. These biochemical characteristics, combined with the presence of endogenous enzymes and naturally occurring microbiota, make shrimp highly susceptible to rapid spoilage during storage. As a consequence, shrimp quality may deteriorate quickly during post-harvest handling and distribution, leading to progressive loss of freshness, development of off-odours, and reduction of sensory acceptability.

The rapid deterioration of seafood products also represents a significant challenge from a sustainability perspective. According to the Food and Agriculture Organization (FAO), approximately 35% of global fishery and aquaculture production is lost or wasted along the supply chain, from harvesting and processing to distribution and retail [3,4]. Highly perishable products such as shrimp are particularly vulnerable to post-harvest losses when storage conditions are suboptimal or when shelf-life estimation is inaccurate. These losses not only generate economic impacts for fisheries and food industries but also represent an inefficient use of natural resources, including marine ecosystems, energy, and labour. Therefore, improving the reliability of freshness evaluation tools is essential not only for quality control and food safety but also for reducing food waste and enhancing the sustainability of seafood supply chains. The deterioration of shrimp quality is a complex and multifactorial process involving both endogenous enzymatic reactions and microbial activity. Immediately after death, autolytic processes dominate and promote the degradation of muscle proteins and nucleotides, leading to the formation of compounds that may subsequently serve as substrates for microbial metabolism [13,14]. As storage progresses, specific spoilage microorganisms (SSOs) become the main drivers of quality deterioration, producing volatile and non-volatile metabolites responsible for sensory defects such as unpleasant odours, flavour changes, and texture degradation [12,15,16,17,18]. Among the microorganisms frequently associated with seafood spoilage during chilled storage are *Pseudomonas* spp., *Shewanella* spp., lactic acid bacteria, *Photobacterium phosphoreum*, and *Brochothrix thermosphacta* [16,17,18,19,20,21,22,23]. The composition and dynamics of the spoilage microbiota may vary depending on several factors, including shrimp species, harvesting area, environmental conditions, and post-harvest handling practices [18,19]. Shrimp can also be contaminated by pathogens such as *E. coli*, *Klebsiella* spp., *Vibrio* spp., *Aeromonas* spp., *Listeria* spp., *Shigella*-like organisms, *Staphylococcus aureus*, and *Salmonella* spp. [24,25]. Their presence depends on environmental conditions in harvesting areas but may also result from improper handling, packaging, storage, or transportation. These microorganisms can contribute not only to spoilage but also to foodborne disease outbreaks [26]. In addition, pathogenic microorganisms can predominate, as observed by Nimiah [27], who found *Aeromons hydrophila* in shrimp and peeled shrimp collected in three local markets in Basrah city.

Because of the complex nature of seafood spoilage, several physicochemical and microbiological parameters have been proposed to evaluate shrimp freshness and quality during storage. Total volatile basic nitrogen (TVB-N) is widely used as an indicator of protein degradation and accumulation of volatile nitrogenous compounds generated during microbial metabolism [28]. Although TVB-N remains a reliable parameter for determining the acceptability of fishery products at the end of shelf life, according to the European Commission Decision 95/149/EC [29], it is not applicable to crustaceans.

Lipid oxidation processes may be monitored through thiobarbituric acid reactive substances (TBARS), while changes in pH can reflect the accumulation of alkaline metabolites during spoilage [30,31]. In addition, compounds derived from the degradation of trimethylamine-N-oxide (TMAO), including formaldehyde and formic acid, have attracted increasing attention as potential indicators of biochemical transformations occurring in marine organisms during post-mortem storage [32,33,34,35,36]. TMAO may be enzymatically demethylated to produce dimethylamine and formaldehyde, and formaldehyde can subsequently be oxidized to formic acid during storage or processing [35,36,37,38]. The concentration of these compounds may therefore provide useful information about the progression of spoilage processes and the biochemical evolution of seafood products.

FA levels vary widely depending on species, size, fishing area, processing, and storage conditions, ranging from 2 to 98 mg/kg [36,39,40,41,42]. Boiling temperature crucially affects FA levels, which decreased during the cooling process by washing with water or changing the roasting practices [41]. FA may decrease during iced storage due to leaching into meltwater or by cooking, although it is not completely removed [42,43,44,45].

However, most available studies have investigated these indicators separately or under controlled laboratory conditions, whereas information integrating microbiological, physicochemical, and sensory parameters under commercial iced storage conditions remains limited. In particular, comprehensive evaluations focusing on Mediterranean shrimp species marketed fresh in retail environments are relatively scarce. A multidimensional assessment combining microbial, chemical, and sensory indicators may therefore provide a more realistic description of shrimp spoilage dynamics and contribute to the identification of practical reference values for freshness classification.

Furthermore, the quality of fresh shrimp is strongly influenced by post-harvest handling practices and by the effectiveness of the cold chain during transport and distribution. After harvesting, shrimp are typically stored in ice and transported to retail markets, where they remain displayed on ice until purchase. Even under refrigerated conditions, however, the metabolic activity of microorganisms and endogenous enzymes continues gradually altering the biochemical composition of muscle tissues. Small variations in storage temperature, handling practices, or hygiene conditions can significantly accelerate spoilage processes, highlighting the importance of reliable indicators capable of monitoring shrimp freshness throughout the commercial distribution chain [12,15].

During chilled storage, the composition of the shrimp microbiota progressively shifts from the initial environmental microbial community to a more specialized group of spoilage microorganisms. These microorganisms, commonly referred to as specific spoilage organisms (SSOs), are responsible for the production of metabolites associated with sensory deterioration, including volatile amines, sulfur-containing compounds, and organic acids [12,16,17,18]. The activity of these microorganisms contributes to the accumulation of compounds such as trimethylamine (TMA), ammonia, and other volatile basic nitrogen compounds that are commonly used as indicators of seafood spoilage [28,46]. Consequently, monitoring the evolution of these chemical compounds provides useful information about the microbial and biochemical processes occurring during storage.

At the same time, biochemical reactions occurring within shrimp tissues can significantly influence the formation and transformation of spoilage-related compounds. The degradation of adenosine triphosphate (ATP) and other non-protein nitrogen compounds generate intermediate metabolites that may influence flavour perception and freshness evaluation. In parallel, the degradation of trimethylamine-N-oxide (TMAO), a compound naturally present in marine organisms, can lead to the formation of dimethylamine and formaldehyde through enzymatic demethylation processes. Formaldehyde may subsequently be oxidized to formic acid during storage or processing, further contributing to the biochemical evolution of seafood products [35,36,37,38].

Understanding the dynamics of these transformations may therefore improve the interpretation of chemical indicators used to evaluate shrimp quality. The concentration of FA and formic acid reflects the extent of TMAO degradation and the spoilage progression [35,36,45,47,48,49,50,51,52]. The European Food Safety Authority (EFSA) has confirmed that most FA detected in fish originates from natural post-mortem biochemical reactions rather than external contamination [49]. The intentional addition of FA is one of the major adulteration practices affecting different types of food categories, including fish and seafood [53,54,55]. High levels of FA in seafood represent a relevant toxicological concern [56,57,58].

From a practical perspective, the relationship between physicochemical indicators and sensory perception is particularly important in determining the actual shelf life of seafood products. While microbial counts and chemical indicators provide objective information about spoilage progression, sensory attributes such as odour, colour, texture, and overall appearance ultimately determine consumer acceptance. For this reason, integrating microbiological, chemical, and sensory evaluations may provide a more realistic and comprehensive assessment of shrimp freshness compared with approaches based on single indicators alone. Such multidimensional evaluations are increasingly recognized as useful tools for improving quality control strategies in seafood processing and distribution systems. Despite numerous studies investigating shrimp spoilage, most available works evaluate individual quality indicators separately or under controlled laboratory conditions. Information integrating microbiological, physicochemical, and sensory parameters under commercial iced storage conditions for Mediterranean shrimp species is absent. Therefore, the aim of the present study was to evaluate the evolution of selected microbiological, physicochemical, and sensory parameters in two commercially important Mediterranean shrimp species (*Parapenaeus longirostris* and *Melicertus kerathurus*) stored on ice under commercial conditions. By analysing the behaviour of these indicators simultaneously, this work aims to contribute to the identification of practical reference ranges that may support shrimp freshness evaluation and shelf-life assessment within seafood supply chains.

## 2. Material and Methods

### 2.1. Sample Collection

A 2-year nationwide survey of fresh and frozen shrimp was conducted. During 2024–2025 fiscal year, 180 samples were collected from local Italian supermarket. The samples included two commercially relevant shrimp species, *P. longirostris* (size 40/kg) and *M. kerathurus* (size 25/kg), harvested in the Mediterranean basin and stored on ice. The collected samples were subjected to microbial and physicochemical analyses on day 0, 4, 8, 12, and 15.

In total, 18 frozen samples of both species were collected from local supermarkets. The samples were thawed in refrigerator at 4 °C for 7 h; then they were stored on ice and analysed to determine formaldehyde concentration at day 0, 4, 8, 12, 15, and 30 days of storage. An additional set of 18 samples was evaluated for formaldehyde content after boiling and frying. Both fresh species samples were boiled in water (100 °C) for 4 min and fried in peanut oil (175 °C) for 4 min. Analyses were conducted in triplicate on three different samples per each sampling point or processing.

### 2.2. Microbiological Analyses

Total mesophilic viable counts (MTVC) and total psychrotrophic viable counts (PTVC) were determined on Plate Count Agar (Oxoid, Milan, Italy) at 30 °C for 48–72 h and 4 °C for 72–96 h, respectively. LAB were enumerated on MRS agar (Oxoid, Milan, Italy) at 30 °C for 48 h. Coagulase-positive staphylococci were enumerated on Baird–Parker agar at 35 °C for 24–48 h; presumptive colonies were confirmed using the tube coagulase test with rabbit plasma containing EDTA. Total coliforms were enumerated on VRBLA (Oxoid, Milan, Italy) at 37 °C for 48 h; *E. coli* on Coli-ID (bioMérieux, Marcy l’Etoile, France) at 37 °C for 48 h; *Pseudomonas* spp. on Pseudomonas Agar Base with CFC (Oxoid, Milan, Italy) at 30 °C for 48 h; sulfite-reducing clostridia in DRCM (VWR, Radnor, PA, USA) at 37 °C for 24–48 h under anaerobiosis (BBL gas-pack system; Becton Dickinson, Franklin Lakjes, NJ, USA); *Vibrio* spp. in Thiosulfate-citrate-bile salts-sucrose agar (TCBS, Oxoid, Milan, Italy) incubated (protected from light) at 37 °C for 18–24 h; and yeasts and moulds on Malt Extract Agar (Oxoid, Milan, Italy) incubated at 25 °C for 3–5 days. *Listeria monocytogenes* and *Salmonella* spp. were investigated according to ISO 11290-1 and ISO 6579-1, respectively [59,60].

### 2.3. Physicochemical Analyses

The pH value was measured using a pH meter (Basic 20; Crison Instruments, Barcelona, Spain) by inserting the pH meter probe into a homogenate obtained by homogenizing 5 g of minced shrimp muscle in 45 mL (1/10) of distilled water [61]. Three calibration buffers were used: pH 4, pH 7, and pH 9. Analysis was made in triplicate on three different samples.

Pearson method [30] was used to evaluate the TVB-N concentration, and Ke et al. [31] method was used to evaluate the oxidation stability during storage (TBARS). Formaldehyde was determined following the method described by Cantoni et al. [36]. Formic acid was detected by Megazyme assay (Neogen; Lansing, MI, USA) according to the manufacturer’s instructions.

### 2.4. Sensory Analysis

Sensory evaluation was performed to assess perceptible differences and quality changes in shrimp samples during iced storage. A total of 20 non-professional assessors (10 women and 10 men, aged 22–30 years), recruited among food technology students and regular seafood consumers, participated in the study. Considering that the assessors were meant to represent the traditional consumers, they were not specifically trained but received preliminary instructions on the evaluation procedure.

Discriminative sensory analysis was carried out using the triangle test, according to ISO 4120 [62]. For each comparison, assessors were presented with three coded samples, two identical and one different, served in randomized order under controlled conditions. Samples from both shrimp species stored on ice for 0, 12, and 15 days were compared as follows: day 0 vs. day 12, day 0 vs. day 15, and day 12 vs. day 15. Assessors were asked to identify the odd sample in each set, indicating whether a perceptible sensory difference was detected.

Following the discriminative assessment, panelists were asked to evaluate the overall sensory quality and acceptability of the samples. Appearance, odour, colour, and texture were considered as integrated attributes. Each sample was rated using a five-point non-parametric ordinal scale, where: 1 = excellent; 2 = very good; 3 = good (acceptable); 4 = poor; and 5 = unacceptable.

Samples were evaluated cold, wrapped in aluminum foil, and presented under standardized lighting conditions. The order of presentation was randomized to minimize order and carryover effects. For each sampling time, 10 samples per shrimp species were evaluated.

Results of the triangle test were evaluated according to the tables and procedures defined in ISO 4120 [62]. Sensory quality scores were treated as ordinal data and analysed descriptively. The sensory methodology followed the principles described by Stone and Sidel [63].

### 2.5. Statistical Analysis

All data were analysed using Statistica software version 7.0 (Statsoft Inc., Tulsa, OK, USA). The values of the different parameters were compared by a one-way analysis of variance, and the means were then compared using the Tukey’s honest significance test. Differences were considered significant at *p* < 0.05.

## 3. Results and Discussion

### 3.1. Microbiological Analysis

At the beginning of storage (day 0), both crustacean species samples exhibited excellent microbiological quality with MTVC and PTVC below 3 log CFU/g (Table 1 and Table 2). As expected, MTVC was higher than PTVC. The investigated crustaceans were harvested in Mediterranean basin, which is an area with a temperate climate, where water temperature is always above 10 °C, favouring the predominance of mesophilic microorganisms.

Various studies have indicated that the initial microbial population depends on the water pH, salinity, pond sediment quality, and temperature [64,65]. In particular, it seems that the salinity and other quality parameters, such as hardness, make shrimp susceptible to pathogens [7]. The consistently low microbial counts observed here suggest that the crustaceans were harvested from areas with favorable water quality. *L. monocytogenes* and *Salmonella* spp. were never detected (absence in 25 g product). *Escherichia coli* and coagulase-positive staphylococci, *Vibrio* spp., and *Clostridium* H_2_S+ were always less than the lower limit of the method (<10 CFU/g). Our results about the initial PTVC and MTVC differ markedly from those reported by other authors, which found values about 5–6 log CFU/g [7,28,66]. Only two studies reported lower initial level of TVC in shrimps of about 2–5 log CFU/g [67] and about 2 CFU/g [68]. In addition, similar MTVC have been reported by Mendes et al. [69] for *P. longirostris*, PTVC by Nirmal and Benjakul [70] for white shrimps, and for blue shrimps by Canizales-Rodriguez et al. [71], demonstrating that the initial level of contamination could be similar even though they are of different species and collected worldwide.

Furthermore, the absence of pathogens demonstrated the good quality of the investigated crustaceans. *Salmonella* spp. and *Listeria monocytogenes* often contaminate both farmed and caught crustaceans. In shrimps, the presence of *Salmonella* spp. and *L. monocytogenes* can vary from 1 to 28% and 1 to 25%, respectively, and it is always at level of presence in 25 g product [25]. Contaminated harvest area and human manipulation may increase the level of pathogens in the product, as demonstrated by Majibur Rahman et al. [24], which found different pathogens such as *E. coli*, *Shigella*, *Salmonella*, *S. aureus*, and *Listeria* at level of 5–7 log CFU/g in shrimp samples collected from hatchery, local markets, and processing plant in Bangladesh.

Conversely, *E. coli* was not detected in the sample collected from all farms by Canizales-Rodriguez et al. [71] and from markets of Dhaka city by Samia et al. [66]; both authors concluded that the sanitary conditions of the shrimp were good in all the investigated sites. It is well known that faecal coliform contents in shrimps vary depending on the sanitary and hygienic conditions of the landing centers, farm, and caught area [72,73]. Their presence is monitored because the presence of faecal coliform and *E. coli* are not allowed in shrimp samples in Japan, USA, and other European countries [74]. Among pathogens, also coagulase-positive staphylococci were not found in both shrimp species analyzed. Their presence mainly depends on human manipulation and is typical of farmed shrimps, as demonstrated by Tawade et al. [7], who detected a contamination level of 10–100 CFU/g. Also, for staphylococci, a lack of hygiene leads to an increase of their concentration, as shown by Samia et al. [66], who found in shrimp samples collected from market around Dhaka city a level of *Staphylococcus* spp. of about 2.7 × 10^5^ CFU/g to 2.3 × 10^7^ CFU/g.

Further proof of the microbial quality of both investigated shrimp species is demonstrated by the low initial concentration of microorganisms responsible for spoilage, such as *Pseudomonas*, Enterobacteriaceae, and yeasts and moulds, which were initially present at levels of 2.2, 1.8, and <10 CFU/g, respectively. These values were lower than those observed by Samia et al. [66], who found 5–7 log CFU/g of *Pseudomonas* spp. in shrimp samples collected from markets around Dhaka city.

However, the microbial growth gradually increased over time in both samples during the cold storage. After 15 days of storage, PTVC, MPTC, *Pseudomonas*, and Enterobacteria reached a concentration level of 6.4, 5.8, 5.5, and 3.9 log CFU/g in *P. longirostris* samples and 6.2, 5.5, 5.6, and 3.9 CFU/g in *M. kerathurus* samples, respectively (Table 1 and Table 2). *E. coli*, coagulase-positive staphylococci, and *Clostridium* H_2_S^+^ concentrations remained below the detection limits of the analytical methods used (<10 CFU/g). On day 0, LAB load was approximately 2 log CFU/g; then it increased and, after 15 days, reached 3.4 log CFU/g. Finally, yeasts and moulds were counted, and after 15 days of storage, they were about 2.2 log CFU/g.

The limited growth of LAB was expected because the samples were stored under aerobic refrigerated conditions, which are unfavorable for lactic acid bacteria, as they are microaerophilic. In fact, they prefer vacuum packaging or modified atmosphere packaging (MAP), where oxygen depletion or CO_2_ enrichment allows their growth favoured by oxygen depletion or CO_2_ enrichment, conditions that occurs during vacuum packaging or MAP [9,15].

Considering the PTVC and MTVC final load after 15 days of storage, both the species samples were acceptable as the generally accepted microbial threshold for seafood spoilage (about 7 log CFU/g) were respected [75]. Conversely, looking at the total coliforms, the samples can be accepted for up to 12 days, when the load is equal or less than 2 log CFU/g. After 15 days, the total coliform load was 3.9 log CFU/g for both the shrimp species, reducing their acceptability, according to the limit proposed by ICMS [75]. Fish and crustacean quality depends primarily on storage temperature, atmosphere composition, initial microbial contamination, and post-harvest handling operations such as gutting, filleting, and packaging [15,26]. There are no specific studies about the microbiological quality of all the commercially crustaceans, and the few indications are often different. Indeed, the FDA [76], which has issued guidelines to define the microbiological quality, indicates in 6 log CFU/g, the microbiological limit for crustacean raw meat. This limit is confirmed by other authors [77,78], which concluded that the total bacterial count at the point of rejection can be around 7–8 log CFU/g. Specifically, considering the level of all investigated microorganisms at the moment of the purchase, both species samples must be considered satisfying according to different authors [24,25,67] and of the recommended Italian guide values of guidelines for official control pursuant to the application of EC Regulations [79,80,81,82,83] and subsequent amendments and additions on microbiological criteria applied to foods (Permanent Conference for Relations between the State and the Regions and the Autonomous Provinces of Trento and Bolzano) [84] and acceptable up to 15 days. In addition, the low initial PTVC and MTVC and the absence of pathogenic and faecal microorganisms indicate that these crustaceans have been manipulated in accordance with the Regulation (EC) No 852/2004 [79] and Regulation (EC) No 853/2004 [80] specific hygiene rules for food of animal origin, including fishery products, and, consequently, must be considered safe.

The acceptability is consistent with literature indicating that fish shelf life depends primarily on storage temperature, atmosphere composition, initial microbial contamination, and post-harvest handling operations such as gutting, filleting, and packaging [9,15]. Consequently, reported shelf-life durations vary considerably among studies. However, our results differ from previous studies [15,85,86], which have reported shorter shelf-life values of about 8 days for crustaceans stored at 2–4 °C in air.

### 3.2. Evolution of pH and TBARS

The pH values and thiobarbituric acid reactive substances (TBARS), an index of lipid oxidation, were initially largely acceptable for both the shrimp species (Table 3).

Then, both pH and TBARS changed during storage. However, despite the increase of the microbial load, they were still acceptable, indicating a good quality of the products. The pH changes due to the endogenous enzymes and microbial activities. Usually, the pH of seafood muscles can vary between 6 and 7 units, depending on the season, seafood species, and handling [71,87].

During the cold storage, endogenous and microbial enzymes produce alkaline metabolites identified as TVB-N [10,36]. The tissue enzymes, mainly adenosine deaminase and adenosine monophosphatase (AMP) deaminase, break down nucleotides and release ammonia, increasing the post-mortem pH of shrimps [46]. Indeed, as shown in Table 3, the pH increased from about 6.8 in *P. longirostris* and 6.9 in *M. kerathurus* on day 0 to 7.4 and 7.5, respectively, after 15 days of storage. On day 0 for both the shrimp species, the pH value was similar to data observed by various authors [88,89] in *Nephros norvegicus* and by Canizales-Rodriguez et al. [71] in blue shrimps. The pH increased significantly during the storage, but the final values for both the shrimp species were lower than those observed by the previously mentioned authors. The final pH values were still acceptable, as they were less of 7.64, indicated as the acceptability limits by Gonglaves et al. [90] for *L. longirostris* stored in ice. Furthermore, in our study, the final pH values were less than 7.8, which is the critical level for shrimp acceptability that different authors have suggested [91,92].

The TBARS values did not significantly change during storage, despite the increase in the microbial load (Table 3). TBARS values increase slightly from 0.6 nmol/g on day 0 to 1.1 nmol/g on day 15 in *P. longirostris* samples and from 0.7 nmol/g on day 0 to 1.0 nmol/g on day 15 in *P. kerathurus*, remaining below the rancidity thresholds. Data were similar to those observed by Angel et al. [93]. Both the shrimp species samples were largely acceptable on 15 days, according to Pearson [30] and Che Man and Ramadas [94], foods are considered non-rancid when TBARS is lower than 8 nmol/g, slightly rancid when is 9–20 nmol/g, and rancid/unacceptable when exceed 21 nmol/g. The change in TBARS is based on the production of malonaldehyde as a result of the dissociation of hydroperoxides formed during fatty acid oxidation. Malonaldehyde is an active compound that can react with various substances, including free amino acids [91]. As free amino acids accumulate during storage at 0 °C, it is possible that condensation took place with these amino acids or even with proteins [95], resulting in lower TBARS values, as was observed in the investigated samples.

### 3.3. Evolution of TVB-N, FA, and Formic Acid

An interesting pattern observed in this study concerns the opposite evolution of formaldehyde and formic acid during iced storage. Formaldehyde concentrations were relatively high in fresh shrimp samples and progressively decreased during storage, while formic acid increased over time. This behaviour is consistent with the biochemical degradation pathway of trimethylamine-N-oxide (TMAO), in which formaldehyde is progressively oxidized to formic acid during post-mortem metabolism.

The simultaneous increase of formic acid, TVB-N, and pH, together with the progressive decline in sensory quality, suggests that these compounds collectively reflect the biochemical progression of shrimp spoilage. This integrated behaviour supports the potential usefulness of formic acid, together with TVB-N, as a complementary indicator of shrimp freshness loss during iced storage.

The evolution of TVB-N, FA, and formic acid values of both the cold-stored shrimp species is reported in Table 4. All the parameters changed significantly during storage (*p* < 0.05), confirming microbial and enzymatic activity as the main drivers of spoilage [11]. Initial TVB-N was 12.9 mg N/100 g in *P. longirostris* and 13.2 mg N/100 in *M. kerathurus* samples; then, after 15 days of storage, it increased progressively to 33.4 mg N/100 g and 34.9 mg N/100 g, respectively. The initial PTVC and MTVC values were less than 3 log CFU/g, and the TVB-N values indicate that both the shrimp species samples were of prime quality because shrimps with a TVB-N less than 18 mg N/100 g were considered excellent [96].

In addition, the final TVB-N concentrations, still below the European Commission limit of 35 mg N/100 g for some fish species [29], demonstrated the acceptability of the shrimps on day 15 of storage. However, as reported by Angel et al. [93], the highest amount of TVB-N for shrimp to be acceptable is 30 mg N/100 g. A similar limit for acceptability (30 mg N/100 g) has been set in Japan and Australia [97]. The TVB-N freshness criteria based on fish values and presented in the European Commission Decision 95/149/EC [29] is therefore considered inappropriate for shrimp species [46].

However, as below demonstrated, the sensory assessment classified as good (usual quality) all the samples evaluated on day 15 of cold storage and regardless of the final TVB-N concentration of shrimps. Indeed, the TVB-N and the correlation with the sensory analysis led us to consider, as followed in other countries [97], that the use of TVB-N as a spoilage indicator in shrimps should follow different degrees of freshness. Mendes et al. [46] suggested levels less than 25 mg N/100 g for fresh shrimps, 25–30 mg/100 g acceptable for consumption, and higher than 30 mg N/100 g as freshness borderline. The initial values and the changes that occurred during storage are comparable to those observed in *P. longirostris* [46,92] and in other shrimp species [98]. Recently, the TVB-N values found by Tawade et al. [7] in cultured shrimp *Litopenaeus vannamei* collected from different farms located in Ratnagiri were similar to those of our investigated samples, and they were lower compared to the results obtained by Puga-Lopez et al. [99]. However, in the literature, it is possible to find some study that demonstrated that freshly caught *L. vannamei* shrimps, collected from farms, could have a TVB-N content of 8.01 mg/100 g [100]. Similarly to TVB-N, FA and formic acid levels changed significantly in both shrimp species samples (*p* < 0.05). These compounds are derived from the degradation of TMAO by the endogenous enzyme TMAO demethylase, producing DMA and FA, part of which is oxidized to formic acid [31]. In this study, initial FA concentrations were 20.3 mg/kg in *P. longirostris* and 18.2 mg/kg in *M. kerathurus* (Table 5), decreasing on day 15 of storage at levels of 1.1 and 0.9 mg/kg, respectively (*p* < 0.05). The decrease is justified by its conversion to formic acid. Indeed, formic acid levels increased steadily throughout storage from 6.1 mg/kg to 17.4 mg/kg in *P. longirostris* and from 5.5 mg/kg to 17.2 mg/kg in *M. kerathurus*, and no significant differences (*p* > 0.05) were found between the two shrimp species. These values are lower than those reported by previous studies [36,40,41] and well below the levels found in other fish species, such as 1.4–4.8 mg/kg in mullet, about 100 mg/kg in cod, and 232–293 mg/kg in deep-frozen hake [34,45,49,50,51,52]. Hoque et al. [101] found FA levels (0.43–2.08 mg/kg) lower than those present in our samples. Moreover, Immaculate and Jamila [102], studying the FA formation in seven fish species, showed that FA levels were significantly higher in non-iced fish compared with the iced fish. The study confirmed that the higher amount of FA results from the natural production during post-mortem enzymatic activity. However, Bianchi et al. [45] studied FA levels in commonly eaten species such as mackerel, trout, sardines, and tuna, founding differences in FA accumulation in freezing fish and generally lower than 25 mg/kg after freezing except for catfish (29.5–53.1 mg/kg). It was concluded that the variation is correlated with endogenous white muscle TMAO demethylase activity [103]. In addition, it can be hypothesised that the FA formation in fish is dependent on muscle type and muscle location [104].

As FA may accumulate naturally through post-mortem degradation, its intentional addition to fresh fish is illegal and poses health risks [41,44,49]. Considering FA a potential carcinogenic compound, EFSA [49] suggests that FA from food of animal and plant origin should not exceed 100–273 mg/kg per day. They estimated that the average dietary exposure was 11 mg/kg per day. Due to the high variability of FA levels, the European Union has not yet established maximum allowable limits in fish and seafood. However, the United States permits FA levels of up to 2 g/kg [54]. The World Health Organization (WHO) has defined a tolerable daily intake (TDI) for FA of 150 μg/kg body weight per day, while the United State Environmental Protection Agency set an ADI of 200 μg/kg of body weight per day [56].

Based on these reference values, FA concentrations measured in both shrimp species fall within acceptable limits. FA levels declined significantly after thawing, from 8.0 to 1.6 mg/kg in *P. longirostris* and from 7.7 to 1.5 mg/kg in *M. kerathurus* (Table 5), probably related to its reduction to formic acid.

Heat treatment further reduced FA concentrations. As shown in Table 6, FA levels decreased by approximately 67% and 80% in *P. longirostris* after boiling and frying, respectively, and by about 69% and 74% in *M. kerathurus*. These results are consistent with those reported by Bhowmik et al. [39], who observed significant reductions in FA levels in fish samples following various pre-treatment and cooking processes. More restrictive limits for FA in seafood have been proposed in Sri Lanka, where regulations do not allow the import, distribution, storage, or sale of seafood containing FA levels exceeding 5 mg/kg [105]. In this study, FA concentrations in all samples did not exceed 5 mg/kg after storage in ice, boiling, or frying.

**Figure 1:**
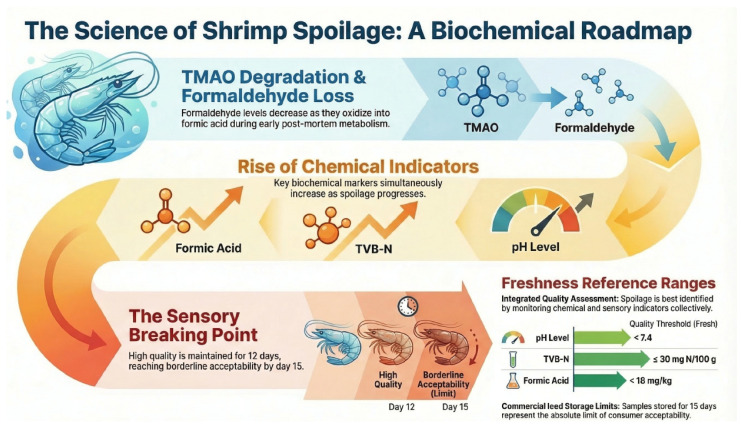
Shrimp freshness evolution.

Finally, within this framework, a set of reference values for key chemical parameters (Figure 1), including pH (<7.4), TVB-N (≤30 mg N/100 g), formaldehyde (<10 mg/kg), and formic acid (<18 mg/kg), is proposed as practical support for shrimp quality classification rather than as regulatory thresholds.

### 3.4. Sensory Analysis

Sensory characteristics play a key role in seafood acceptance and are therefore essential parameters when evaluating the effectiveness of preservation methods [92]. For this reason, shrimp samples were evaluated by 20 non-professional assessors to investigate perceptible differences and changes in overall quality during iced storage.

Triangle test results showed that clear and statistically significant sensory differences (*p* < 0.05) were detected when comparing samples stored for 0 vs. 15 days and 12 vs. 15 days for both *Parapenaeus longirostris* and *Melicertus kerathurus* (Table 7). In these comparisons, all assessors correctly identified the odd sample, largely exceeding the minimum number of correct responses required for significance according to ISO 4120 [62]. These results indicate that storage for 15 days on ice led to perceptible sensory changes, mainly related to odour and overall freshness perception.

In contrast, comparisons between samples stored for 0 and 12 days did not reveal statistically significant differences (*p* > 0.05). Although some assessors reported perceiving slight differences, the number of correct identifications (8/20 for *P. longirostris* and 10/20 for *M. kerathurus*) did not reach the threshold required for significance. This suggests that sensory modifications occurring within the first 12 days of iced storage were limited and not consistently detectable.

Despite the absence of significant discriminative differences between samples stored for 0 and 12 days, all samples were judged acceptable. Samples evaluated at day 0 were predominantly rated as excellent, while those stored for 12 days were generally rated as very good, confirming that iced storage effectively preserved sensory quality during this period.

Samples stored for 15 days, although still considered acceptable, were mostly rated as good, indicating a perceptible decline in sensory quality and positioning these samples close to the limit of consumer acceptability. This borderline sensory condition is consistent with the physico-chemical trends observed during storage and supports the definition of a practical shelf-life limit for both shrimp species under the applied storage conditions.

Overall, the combined discriminative and acceptability results indicate a progressive sensory quality deterioration with storage time, with 12 days representing a phase of high sensory acceptability and 15 days marking the onset of quality loss perceivable by consumers.

The sensory quality evolution observed during iced storage was closely associated with the behaviour of the main chemical and physicochemical indicators of freshness and spoilage. In both *Parapenaeus longirostris* and *Melicertus kerathurus*, samples stored for up to 12 days, which were predominantly rated as excellent or very good by the assessors, showed TVB-N values below 30 mg N/100 g, pH values ranging between 6.8 and 7.2, low TBARS values (≤0.9 nmol malonaldehyde/g), and moderate concentrations of formic acid. These conditions are indicative of limited protein degradation and oxidative processes and are consistent with a high sensory perception of freshness.

Conversely, samples stored for 15 days, which were still considered acceptable but rated as good and close to the sensory acceptability limit, exhibited TVB-N values exceeding 30 mg N/100 g, a further increase in pH up to 7.4–7.5, and formic acid concentrations approaching 17 mg/kg in both shrimp species. The concomitant accumulation of volatile basic compounds and acidic metabolites is known to contribute to the development of off-odours and loss of freshness, coherently reflecting the sensory changes perceived by the assessors at this storage time.

Formaldehyde showed a distinct pattern, with relatively high concentrations in fresh samples followed by a rapid decrease during iced storage, reaching values close to 1 mg/kg at the end of the storage period. This behaviour, together with the marked reduction observed after thermal treatments (boiling and frying), indicates that formaldehyde levels in raw shrimp should be interpreted primarily as a freshness-related parameter rather than a direct determinant of sensory rejection. Indeed, the decline in formaldehyde content did not negatively affect sensory acceptability, which remained high up to 12 days of storage.

The progressive decline of the biochemical freshness index observed during the first hours of storage further supports the onset of biochemical changes preceding sensory perception, which become evident only after prolonged storage. Overall, the strong agreement between sensory evaluation and chemical indicators supports a multidimensional assessment of shrimp shelf life, indicating that up to 12 days of iced storage ensures high sensory quality, while 15 days represents a borderline condition in which chemical indicators and sensory perception converge towards the limit of acceptability.

## 4. Conclusions

The results demonstrate that shrimp quality during iced storage is governed by the combined action of microbial growth and post-mortem biochemical processes. Sensory evaluation showed that high product quality was maintained for approximately 12 days, while storage for 15 days represented a borderline acceptability condition. The two shrimp species investigated showed comparable behaviour under iced storage, indicating that common freshness indicators can be applied across species.

The integrated interpretation of chemical, microbiological, and sensory indicators provides a useful framework for shrimp shelf-life evaluation under commercial conditions. The reference ranges proposed in this study may support quality control strategies within seafood supply chains and contribute to reducing unnecessary food waste by improving the reliability of freshness assessment.

Among the investigated indicators, the opposite evolution of formaldehyde and formic acid during storage appears particularly informative, reflecting the biochemical progression of shrimp spoilage and supporting the usefulness of formic acid as a complementary freshness indicator.

## Figures and Tables

**Table 1:** Evolution of microorganisms in *Parapenaeus longirostris* stored on ice.

*Parapenaeus longirostris*	Days				
Microorganisms	0	4	8	12	15
Mesophilic TVC	2.5 ± 0.5 ^a^	4.1 ± 0.3 ^b^	5.1 ± 0.3 ^c^	5.7 ± 0.1 ^d^	5.8 ± 0.2 ^d^
Psycrophilic TVC	1.9 ± 0.2 ^a^	2.9 ± 0.1 ^b^	5.8 ± 0.5 ^c^	6.2 ± 0.2 ^c^	6.4 ± 0.4 ^c^
Total Coliforms	1.5 ± 0.1 ^a^	1.6 ± 0.2 ^a^	1.8 ± 0.3 ^a^	2.0 ± 0.1 ^b^	3.9 ± 0.3 ^c^
*Pseudomonas* spp.	2.5 ± 0.2 ^a^	3.8 ± 0.2 ^b^	4.2 ± 0.1 ^c^	4.9 ± 0.1 ^d^	5.5 ± 0.4 ^e^
*E. coli*	<10	<10	<10	<10	<10
*Staphylococcus* coagulase +	<10	<10	<10	<10	<10
*Vibrio* spp.	<10	<10	<10	<10	<10
Lactic acid bacteria	2.0 ± 0.1 ^a^	2.1 ± 0.2 ^a^	2.8 ± 0.1 ^b^	2.9 ± 0.2 ^b^	3.3 ± 0.1 ^b^
Yeast and moulds	<10 ^a^	<10 ^a^	2.0 ± 0.1 ^b^	2.3 ± 0.3 ^b^	2.5 ± 0.5 ^b^
Clostridia H_2_S+	<10	<10	<10	<10	<10

Data represent the range and means ± standard deviations of the total samples; mean with the same letters within a lane (following the values), considering each single parameter is not significantly different (*p* < 0.05). Analyses were conducted in triplicate on three different samples per each sampling point. Data log CFU/g; <10 CFU/g.

**Table 2:** Evolution of microorganisms in *Melicertus kerathurus* stored in ice.

*Melicertus kerathurus*	Days				
Microorganisms	0	4	8	12	15
Mesophilic TVC	2.8 ± 0.1 ^a^	3.9 ± 0.3 ^b^	4.2 ± 0.1 ^b^	4.8 ± 0.3 ^c^	5.4 ± 0.3 ^c^
Psycrophilic TVC	2.0 ± 0.1 ^a^	2.6 ± 0.3 ^b^	4.7 ± 0.2 ^c^	5.5 ± 0.1 ^d^	6.2 ± 0.2 ^e^
Total Coliforms	1.8 ± 0.2 ^a^	1.8 ± 0.1 ^a^	2.0 ± 0.1 ^b^	2.0 ± 0.2 ^b^	3.9 ± 0.1 ^c^
*Pseudomonas* spp.	2.2 ± 0.1 ^a^	3.5 ± 0.3 ^b^	4.4 ± 0.1 ^c^	4.8 ± 0.2 ^d^	5.6 ± 0.2 ^e^
*E. coli*	<10	<10	<10	<10	<10
*Staphylococcus* coagulase +	<10	<10	<10	<10	<10
*Vibrio* spp.	<10	<10	<10	<10	<10
Lactic acid bacteria	2.0 ± 0.2 ^a^	2.3 ± 0.1 ^a^	2.7 ± 0.2 ^b^	3.0 ± 0.3 ^b^	3.4 ± 0.1 ^b^
Yeast and moulds	<10 ^a^	<10 ^a^	2.0 ± 0.2 ^b^	2.1 ± 0.1 ^b^	2.2 ± 0.3 ^b^
Clostridia H_2_S+	<10	<10	<10	<10	<10

Data represent means ± standard deviations of the total samples; mean with the same letters within a lane (following the values), considering each single parameter is not significantly different (*p* < 0.05). Analyses were conducted in triplicate on three different samples per each sampling point. Data log CFU/g; <10 CFU/g.

**Table 3:** Changes of pH and TBARS in crustacean species stored on ice.

Days	*Parapenaeus longirostris*		*Melicertus kerathurus*	
	pH	TBARS	pH	TBARS
0	6.8 ± 0.2 ^a^	0.6 ± 0.3 ^a^	6.8 ± 0.2 ^a^	0.7 ± 0.1 ^a^
4	6.9 ± 0.1 ^a^	0.6 ± 0.3 ^a^	6.8 ± 0.1 ^a^	0.8 ± 0.2 ^a^
8	6.9 ± 0.1 ^a^	0.9 ± 0.1 ^a^	7.0 ± 0.1 ^b^	0.8 ± 0.2 ^a^
12	7.1 ± 0.1 ^b^	0.9 ± 0.2 ^a^	7.2 ± 0.1 ^b^	0.9 ± 0.1 ^a^
15	7.4 ± 0.1 ^c^	1.1 ± 0.1 ^a^	7.5 ± 0.1 ^c^	1.0 ± 0.1 ^a^

Data represent the range and means ± standard deviations of the total samples; mean with the same letters within each column (following the values), considering each single parameter is not significantly different (*p* < 0.05). Analyses were conducted in triplicate on three different samples per each sampling point. TBARS: nmol malonaldehyde/g.

**Table 4:** Evolution of TVB-N, formaldehyde, and formic acid in crustacean species stored on ice.

	*Parapenaeus longirostris*			*Melicertus kerathurus*		
Days	TVB-N	Formaldehyde	Formic Acid	TVB-N	Formaldehyde	Formic Acid
0	12.9 ± 0.8 ^a^	20.3 ± 1.8 ^f^	6.1 ± 2.2 ^k^	13.2 ± 1.1 ^a^	18.2 ± 2.1 ^f^	5.6 ± 2.2 ^k^
4	15.5 ± 0.5 ^b^	4.8 ± 2.1 ^g^	12.9 ± 2.1 ^l^	19.2 ± 0.8 ^b^	4.9 ± 1.2 ^g^	14.5 ± 0.7 ^l^
8	22.4 ± 0.3 ^c^	1.5 ± 1.3 ^g^	13.8 ± 3.3 ^l^	23.3 ± 1.1 ^c^	3.3 ± 1.1 ^g^	13.5 ± 1.0 ^l^
12	27.8 ± 1.1 ^d^	1.3 ± 0.4 ^g^	15.5 ± 1.8 ^l^	29.2 ± 0.2 ^d^	1.2 ± 0.3 ^h^	15.4 ± 0.8 ^m^
15	33.4 ± 0.3 ^e^	1.1 ± 0.3 ^g^	17.4 ± 1.3 ^l^	34.9 ± 0.1 ^e^	0.9 ± 0.2 ^h^	17.2 ± 0.9 ^n^

Data represent means ± standard deviations of the total samples; mean of TVB-N (^a–e^), formaldehyde (^f–h^), and formic acid (^k–n^) with the same letters within each column (following the values), considering each single parameter is not significantly different (*p* < 0.05). Analyses were conducted in triplicate on three different samples per each sampling point. Formaldehyde and formic acid: mg/kg; TVB-N: mg N/100 g.

**Table 5:** Evaluation of formaldehyde (mg/kg) in crustaceans thawed at 2 °C for 7 h and storage on ice up to 30 days.

Days	*Parapenaeus longirostris*	*Melicertus kerathurus*
0	8.0 ± 0.5 ^a^	7.7 ± 0.2 ^a^
4	7.2 ± 0.2 ^a^	6.9 ± 0.3 ^b^
8	4.8 ± 0.1 ^b^	4.6 ± 0.1 ^c^
12	3.2 ± 0.3 ^c^	3.1 ± 0.4 ^d^
15	2.8 ± 0.5 ^c^	2.7 ± 0.2 ^d^
30	1.6 ± 0.6 ^d^	1.5 ± 0.3 ^e^

Data represent means ± standard deviations of the total samples; mean with the same letters within each species and column (following the values), considering each single parameter is not significantly different (*p* < 0.05). Analyses were conducted in triplicate on three different samples per each sampling point.

**Table 6:** Formaldehyde residue in crustaceans after boiling (100 °C for 4 min) and frying (175 °C for 4 min).

Samples	Formaldehyde (mg/kg)	
	*Parapenaeus longirostris*	*Melicertus kerathurus*
Control	23.5 ± 3.1 ^aA^	25.3 ± 2.7 ^aA^
Boiled	7.8 ± 0.2 ^aB^	8.0 ± 0.1 ^aB^
Fried	4.7 ± 1.5 ^aB^	6.3 ± 2.3 ^aB^

Data represent means ± standard deviations of the total samples; mean with the same lowercase letters within a lane (following the lanes) and the same uppercase letters within each column, considering each single parameter is not significantly different (*p* < 0.05). Analyses were conducted in triplicate on three different samples per each sampling point.

**Table 7:** Sensory discriminative and quality evaluation of shrimp samples during storage on ice.

Shrimp Species	Storage Time Comparison	Correct Responses (n/20)	Triangle Test Significance	Predominant Quality Score *	Overall Acceptability
*Parapenaeus longirostris*	0 vs. 12 days	8	Not significant (*p* > 0.05)	1 vs. 2	Acceptable
	0 vs. 15 days	20	Significant (*p* < 0.05)	1 vs. 3	Acceptable
	12 vs. 15 days	20	Significant (*p* < 0.05)	2 vs. 3	Acceptable
*Melicertus kerathurus*	0 vs. 12 days	10	Not significant (*p* > 0.05)	1 vs. 2	Acceptable
	0 vs. 15 days	20	Significant (*p* < 0.05)	1 vs. 3	Acceptable
	12 vs. 15 days	20	Significant (*p* < 0.05)	2 vs. 3	Acceptable

* Quality score scale: 1 = excellent; 2 = very good; 3 = good (acceptable); 4 = poor; 5 = unacceptable.

## Data Availability

The data in this study are readily available upon reasonable request to the corresponding authors.

## References

[1] Aydın C.M., Tıraşın E.M., Ünlüoğlu A., Dal İ., Akalın S., Karakuş M. (2025). Population dynamics of the deep-water rose shrimp *Parapenaeus longirostris* (Lucas, 1846) in Antalya Bay (eastern Mediterranean Sea, southern Türkiye). Reg. Stud. Mar. Sci..

[2] Chatzispyrou A., Lampri P.N., Fytilakos I., Kapiris K. (2018). Morphological study of the deep-water rose shrimp *Parapenaeus longirostris* (Lucas, 1846) (Crustacea: Decapoda: Dendrobranchiata: Penaeidae) in the eastern Ionian and Aegean Sea (Eastern Mediterranean Sea). Cah. Biol. Mar..

[3] FAO GLOBEFISH (2025). Quarterly Shrimp Analysis—October 2025.

[4] FAO (2025). General Fisheries Commission for the Mediterranean—Data Collection Reference Framework (DCRF).

[5] Heu M.S., Kim J.S., Shahidi F. (2003). Components and nutritional quality of shrimp processing by-products. Food Chem..

[6] Oksuz A., Ozyilmaz A., Aktas M., Gercek G., Motte J. (2009). A comparative study on proximate, mineral and fatty acid compositions of deep seawater rose shrimp (*Parapenaeus longirostris*, Lucas 1846) and red shrimp (*Plesionika martia*, A. Milne-Edwards, 1883). J. Anim. Vet. Adv..

[7] Tawade P.S., Koli J.M., Patange S.B., Sharangdher S.T., Mohite A.S. (2019). Biochemical and Microbiological Quality of Cultured Shrimp *Litopenaeus vannamei* of Different Farms of Ratnagiri. Int. J. Curr. Microbiol. Appl. Sci..

[8] Seafood Market Reports. https://www.reportlinker.com/market-report/Seafood/4209/Seafood.

[9] Pellegrini M., Andyanto D., Iacumin L., Comi G. (2025). Evaluation of Total Volatile Basic Nitrogen, Formaldehyde, and Formic Acid as Markers to Define the Acceptability of Farmed Sea Bass and Sea Bream Stored Under Vacuum (VP) or in Modified-Atmosphere Packaging (MAP) at 4 ± 2 °C. Microorganisms.

[10] Huss H.H. (1995). Quality and Quality Changes in Fresh Fish.

[11] Comi G., Iacumin L., Bevilacqua A., Corbo M.R., Sinigaglia M. (2024). Meat and Fish spoilage. The Microbial Quality of Food.

[12] Dalgaard P. (1995). Qualitative and quantitative characterization of spoilage bacteria from packed fish. Int. J. Food Microbiol..

[13] Carrascosa C., Millian R., Saavedra P., Jaber J.R., Raposo A., Peréz E., Montenegro T., Sanjuán E. (2015). Microbiological evolution of gilthead sea bream (*Sparus aurata*) in Canary Islands during ice storage. J. Food Sci. Technol..

[14] Bassi L.E., Hassouna M., Shinzato N., Matsgui T. (2009). Biopreservation of Refrigerated and Vacuum-Packed *Dicentrarchus labrax* by Lactic Acid Bacteria. J. Food Sci..

[15] Declerck D., van Hauwaert H. (1981). Value of the Indol Determination as an Objective Quality Test on Commercial Shrimps; Mededelingen van het Rijksstation voor Zeevisserij (Belgium). no. 174.

[16] Gram L., Huss H.H. (1996). Microbiological spoilage of fish and fish products. Int. J. Food Microbiol..

[17] Liston J., Connell J.J. (1980). Microbiology in fishery science. Advances in Fish Science and Technology.

[18] Broekaert K., Heyndrickx M., Herman L., Devlieghere F., Vlaemynck G. (2011). Seafood quality analysis: Molecular identification of dominant microbiota after ice storage on several general growth media. Food Microbiol..

[19] Chinivasagam H.N., Bremner H.A., Thrower S.J., Nottingham S.M. (1996). Spoilage pattern of five species of Australian prawns: Deterioration is influenced by environment of capture and mode of storage. J. Aquat. Food Prod. Technol..

[20] Mejlholm O., Boknaes N., Dalgaard P. (2005). Shelf life and safety aspects of chilled cooked and peeled shrimps (*Pandalus borealis*) in modified atmosphere packaging. J. Appl. Microbiol..

[21] Noseda B., Goethals J., De Smedt L., Dewulf J., Samapundo S., Van Langenhove H., Devlieghere F. (2012). Effect of O_2_ e CO_2_ enriched atmospheres on microbiological growth and volatile metabolite production in packaged cooked peeled gray shrimp (*Crangon crangon*). Int. J. Food Microbiol..

[22] Yang S.P., Xie J., Qian Y.F. (2017). Determination of Spoilage Microbiota of Pacific White Shrimp During Ambient and Cold Storage Using Next-Generation Sequencing and Culture-Dependent Method. J. Food Sci..

[23] Qian Y.F., Yang S.P., Xie J., Xiong Q., Gao Z.L. (2013). Impact of the O_2_ concentrations on bacterial communities and quality of modified atmosphere packaged Pacific white shrimp (*Litopenaeus vannamei*). J. Food Sci..

[24] Majibur Rahman M., Rahman F., Afroze F., Yesmin F., Fatema K.K., Kanta Das K., Noor R. (2012). Prevalence of Pathogenic Bacteria in Shrimp Samples Collected from Hatchery, Local Markets and the Shrimp Processing Plant. Bangladesh J. Microbiol..

[25] Gecan J.S., Blander R., Staruszkiewicz W.F. (1994). Fresh and Frozen Shrimp: A profile of filth, Microbiological Contamination, and Decomposition. J. Food Prot..

[26] Wallace B.J., Guzewich J.J., Cambridge M., Altekruse S., Morse D.L. (1999). Seafood-Associated Disease outbreaks in New York, 1980–1994. Am. J. Prev. Med..

[27] Niamah A.K. (2012). Detected of aerogene in *Aeromonas hydrophila* isolates from shrimp and peeled shrimp samples in local markets. J. Microbiol. Biotech. Food Sci..

[28] Macé S., Cardinal C., Jaffrès E., Cornet J., Lalanne V., Chevalier F., Sérot T., Pilet M.F., Dousset X., Joffraud J.J. (2014). Evaluation of the spoilage potential of bacteria isolated from spoiled cooked whole tropical shrimp (*Penaeus vannamei*) stored under modified atmosphere packaging. Food Microbiol..

[29] European Commission (1995). EEC/95—Commission Decision of 8 March 1995 fixing the total volatile basic nitrogen (TVB-N) limit values for certain categories of fishery products and specifying the analysis methods to be used (95/149/EC). No. L 97/84, 29/04/1995. Off. J. Eur. Communities.

[30] Pearson D. (1973). Laboratory Techniques in Food Analysis.

[31] Ke P.Y., Cervantes E., Robles-Martınez C. (1984). Determination of thiobarbituric acid reactive substances (TBARS) in fish tissue by an improved distillation spectrophotometer method. J. Sci. Food Agric..

[32] Tappi S., De Aguiar Saldanha Pinheiro A.C., Mercatante D., Picone G., Soglia F., Rodriguez-Estrada M.T., Rocculi P. (2020). Quality changes during frozen storage of mechanical-separated flesh obtained from an underutilized crustacean. Foods.

[33] Parlapani F.F., Haroutounian S.A., Nychas G.J.E., Boziaris I.S. (2015). Microbiological spoilage and volatiles production of gutted European sea bass stored under air and commercial modified atmosphere package at 2 °C. Food Microbiol..

[34] Dainty R.H. (1996). Chemical/biochemical detection of spoilage. Int. J. Food Microbiol..

[35] Summers G., Wibisono R.D., Hedderley D.I., Fletcher G.C. (2017). Trimethylamine oxide content and spoilage potential of NewZealand commercial fish species. N. Z. J. Mar. Freshw. Res..

[36] Cantoni C., Renon P., Comi G. (1978). Trasformazione dell’aldeide formica nelle carni di animali marini. Arch. Vet. Ital..

[37] Fu X., Xue C., Miao B., Liang J., Li Z., Cui F. (2006). Purification and characterization of trimethylamine-N-oxide demethylase from jumbo squid (*Dosidicus gigas*). J. Agric. Food Chem..

[38] Benjakul S., Visessanguan W., Tanaka M. (2003). Partial purification and characterization of trimethylamine-N-oxide demethylase from lizardfish kidney. Comp. Biochem. Physiol. Part B Biochem. Mol. Biol..

[39] Bhowmik S., Begum M., Alam A.K.M.N. (2020). Formaldehyde Associated risk assessment of fish sold in local markets of Bangladesh. Agric. Res..

[40] Cantoni C., Bianchi M.A., Beretta G. (1976). L’aldeide formica nei prodotti ittici. Arch. Vet. It..

[41] Jinadasa B.K.K.K., Elliott C., Jayasinghe G.D.T.M. (2022). A review of the presence of formaldehyde in fish and seafood. Food Control.

[42] Sanyal S., Sinha K., Saha S., Banerjee S. (2017). Formalin in fish trading: An inefficient practice for sustaining fish quality. Fish. Aquat. Life.

[43] Laly S., Priya E., Panda S., Zynudheen A. (2018). Formaldehyde in seafood: A review. Fish. Technol..

[44] Aminah A.S., Zailina H., Fatimah A.B. (2013). Health risk assessment of adults consuming commercial fish contaminated with formaldehyde. Food Public Health.

[45] Bianchi F., Careri M., Musci M., Mangia A. (2007). Fish and food safety: Determination of formaldehyde in 12 fish species by SPME extraction and GC–MS analysis. Food Chem..

[46] Mendes R., Goncalves A., Pestana J., Pestana C. (2005). Indole production and deepwater pink shrimp (*Parapenaeus longirostris*) decomposition. Eur. Food Res. Technol..

[47] Yeh T.S., Lin T.C., Chen C.C., Wen H.M. (2013). Analysis of free and bound formaldehyde in squid and squid products by gas chromatography–mass spectrometry. J. Food Drug Anal..

[48] Chung S.W.C., Chan B.T.P. (2009). Trimethylamine oxide, dimethylamine, trimethylamine and formaldehyde levels in main traded fish species in Hong Kong. Food Addit. Contam. Part B..

[49] European Food Safety Authority (2014). Endogenous formaldehyde turnover in humans compared with exogenous contribution from food sources. Eur. Food Saf. Auth. J..

[50] Anissah U., Putri A.K., Barokah G.R. (2019). An estimation of endogenous formaldehyde exposure due to consumption of Indonesian opah fish (*Lampris guttatus*) in three major export destination countries. Squalen Bull. Mar. Fish. Postharvest Biotechnol..

[51] Chandralekha A., Baranage C., Samarajeewa U. (2017). Formaldehyde levels in fish from the Kandy market. J. Nation. Sci. Found. Sri Lanka.

[52] Devaraj P., Babu V., Cengiz E.I. (2021). Qualitative detection of formaldehyde and ammonia in fish and other seafoods obtained from Chennai’s (India) fish markets. Environ. Monit. Assess..

[53] Zhu J., Li J., Jia J. (2012). Effects of thermal processing and various chemical substances on formaldehyde and dimethylamine formation in squid *Dosidicus gigas*. J. Sci. Food Agric..

[54] Wahed P., Razzaq M.A., Dharmapuri S., Corrales M. (2016). Determination of formaldehyde in food and feed by an in-house validated HPLC method. Food Chem..

[55] Kundu M., Prasad S., Krishnan P., Gajjala S. (2019). A novel electrochemical biosensor based on hematite (α-Fe_2_O_3_) flowerlike nanostructures for sensitive determination of formaldehyde adulteration in fruit juices. Food Bioprocess Technol..

[56] USEPA (1991). Formaldehyde.

[57] Pearce N., Blair A., Vineis P., Ahrens W., Andersen A., Anto J.M., Armstrong B.K., Baccarelli A.A., Beland F.A., Berrington A. (2015). IARC monographs: 40 years of evaluating carcinogenic hazards to humans. Environ. Health Perspect..

[58] U.S. Department of Health and Human Services (2020). Report on Carcinogens.

[59] (2004). Microbiology of Food and Animal Feeding Stuffs—Horizontal Method for the Detection of Listeria monocytogenes..

[60] (2004). Microbiology of Food and Animal Feeding Stuffs—Horizontal Method for the Detection of Salmonella spp.

[61] Boland F.E., Lin R.C., Mulvaney T.R., MCclure F.D., Johnston M.R. (1981). pH Determination in Acidified Foods: Collaborative Study. J. Assoc. Off. Anal. Chem..

[62] (2004). Triangle Test Methodology. Standard Test Method for Sensory Analysis—General Guidance for the Design of Test Rooms.

[63] Stone H., Sidel J.L. (2004). Sensory Evaluation Practices.

[64] Lo C.f., Kou G.H. (1998). Virus-associated white spot syndrome of shrimp in Taiwan, a review. Fish Pathol..

[65] Takahashi Y., Shimoyama Y., Momoyama K. (1985). Pathogenicity and characteristics of *Vibrio* sp. isolated from cultured kuruma prawn *Penaeus japonicus* Bate. Bull. Jpn. Soc. Sci. Fish..

[66] Samia S., Galib H.T., Tanvir A.S., Basudeb C.S., Walliullah M., Tasnia A., Mrityunjoy A. (2014). Microbiological quality analysis of shrimps collected from local market around Dhaka city. Int. Food Res. J..

[67] Yousuf A.H.M., Ahmed M.K., Yeasmin S., Ahsan N., Rahman M.M., Islam M.M. (2008). Prevalence of microbial load in shrimp, *Penaeus monodon* and prawn, *Macrobrachium rosenbergii* from Bangladesh. World J. Agric. Sci..

[68] Zapatka F., Bartolomeo B. (1973). Microbiological Evaluation of Cold-Water Shrimp (*Pandalus borealis*). Appl. Microbiol..

[69] Mendes R., Quinta R., Nunes M.L. (2001). Changes in baseline levels of nucleotides during ice storage of fish and crustaceans from the Portuguese coast. Eur. Food Res. Technol..

[70] Nirmal N.P., Benjakul S. (2009). Effect of ferulic acid on inhibition of polyphenoloxidase and quality changes of Pacific white shrimp (*Litopenaeus vannamen*) during iced storage. Food Chem..

[71] Canizales-Rodriguez D.F., Ocano-Higuera V.M., Marquez-Rios E., Graciano-Verdugo A.Z., Càrdena-Lopez J.L., Yepiz-Gòmez M.S. (2015). Biochemical, Physical, Chemical, and Microbiological assessment of Blue Shrimp (*Litopenaeus stylirostris*) stored in ice. J. Aquat. Food Prod. Technol..

[72] Ali S.S.R., Ramachandran M., Chakma S.K., Asrar M. (2017). Proximate composition of commercially important marine fishes and shrimps from the Chennai coast, India. Int. J. Fish. Aquat. Stud..

[73] Hossain A., Mandal S.C., Rahman M.S., Rahman M.M., Hasan M. (2010). Microbiological quality of processed frozen black tiger shrimps in fish processing plant. World J. Fish Mar. Sci..

[74] Azzam M., Sekheta F., Sahtout A.H., Hisham A., Sekheta F., Sharabi R.O., Airoud K.A. (2010). The group of hidden hazards in enhanced HACCP and ISO-22000 based quality systems. Int. J. Food Saf..

[75] ICMS, International Commission on Microbiological Specification for Foods (1986). Microorganisms in Foods Sampling for Microbiological Analysis: Principles and Specific Applications.

[76] FDA (2013). Revised Guidelines for the Assessment of Microbiological Quality of Processed Food.

[77] Espe M., Kiessling A., Lunestad B.T., Torrissen O.J., Rørå A.M.B. (2004). Quality of cold smoked salmon collected in one French hypermarket during a period of 1 year. Food Sci. Technol..

[78] Olafsdottir G., Chanie E., Westad F., Luten J., Kristbergsson K. (2005). Prediction of microbial and sensory quality of cold smoked atlantic salmon *Salmo salar* by electronic nose. J. Food Sci..

[79] Regulation (EC) No 852/2004 of the European Parliament and of the Council of 29 April 2004 on the Hygiene of Foodstuffs. http://data.europa.eu/eli/reg/2004/852/oj.

[80] Regulation (EC) No 853/2004 of the European Parliament and of the Council of 29 April 2004 Laying Down Specific Hygiene Rules for Food of Animal Origin. http://data.europa.eu/eli/reg/2004/853/oj.

[81] Commission Regulation (EC) No 2073/2005 of 15 November 2005 on Microbiological Criteria for Foodstuffs (Text with EEA Relevance). http://data.europa.eu/eli/reg/2005/2073/oj.

[82] Regulation (EC) No 882/2004 of the European Parliament and of the Council of 29 April 2004 on Official Controls Performed to Ensure the Verification of Compliance with Feed and Food Law, Animal Health and Animal Welfare Rules. http://data.europa.eu/eli/reg/2004/882/oj.

[83] Regulation (EC) No 854/2004 of the European Parliament and of the Council of 29 April 2004 Laying Down Specific Rules for the Organisation of Official Controls on Products of Animal Origin Intended for Human Consumption. http://data.europa.eu/eli/reg/2004/854/oj.

[84] Linee Guida per il Controllo Ufficiale ai Sensi dei Regolamenti (CE) 882/2004 e 854/2004” Repertorio Atti n. 212-CSR del 10/11/2016. https://www.iss.it/cnr-lpbl-normativa/-/asset_publisher/hCdLcO9sbUko/content/normativa-1-1.

[85] Noseda B., Vermeulen A., Ragaert P., Devlieghere F., Boziaris I.S. (2014). Packaging of fish and fishery products. IFST Advances in Food Science Series. Seafood Processing: Technology, Quality & Safety.

[86] Kostaki M., Giatrakou V., Savvaidis I.N., Kontominas M.G. (2009). Combined effect of MAP and thyme essential oil on the microbiological, chemical and sensory attributes of organically aquacultured sea bass (*Dicentrarchus labrax*) fillets. Food Microbiol..

[87] Simeonidou S., Govans A., Kawabata M. (1998). Quality assessment of seven Mediterranean fish species during storage. Food Res. Int..

[88] Martinez O.A., Gòmez G.M.C., Montero P. (2008). Chemical and microbial indexes of Norvegian lobsters (*Nephros norvegicus*) dusted with sulphites. J. Food Sci..

[89] Huidobro A., López-Caballero M., Mendes R. (2002). Onboard processing of deepwater pink shrimp (Parapenaeus longirostris) with liquid ice: Effect on quality. Eur. Food Res. Technol..

[90] Gonglaves A.C., Lopez-Caballero M.E., Nunes M.L. (2003). Quality changes of deepwater pink shrimp (*Parapenaeus longirostris*) packed in modified atmosphere. J. Food Sci..

[91] Chung C.Y., Lain J.L. (1979). Study on the composition of frozen shrimp II. Deterioration during iced and refrigerated storage. Nat. Sci. Counc. Mon. (ROC).

[92] Mendes R., Huidobro A., Lopez-Caballero E. (2002). Indole levels in deepwater pink shrimp (*Parapenaeus longirostris*) from the Portuguese coast. Eff. Temp. Abuse. Eur. Food Res. Technol..

[93] Angel S., Basker D., Kanner J., Juven B.J. (1981). Assessment of shelf-life of fresh water prawns stored at 0 °C. J. Food Technol..

[94] Che Man Y.B., Ramadas J. (1998). Effect of packaging environment on quality changes of smoked Spanish mackerel under refrigeration. J. Food Qual..

[95] Kanner J., Karel M. (1976). Changes in lysozyme due to reactions with peroxidizing methyl linoleate in a dehydrated model system. J. Agric. Food Chem..

[96] Chang O., Cheuk W.L., Nickelson R., Martin R., Finne G. (1983). Indole in shrimp: Effect of fresh storage temperature, freezing and boiling. J. Food Sci..

[97] Chambers T.L., Staruszkiewicz A.W. (1981). Ultraviolet detection procedure for liquid chromatographic determination of indole in shrimp. J. Assoc. Off. Anal. Chem..

[98] Iyengar J.R., Visweswariah K., Moorjani M.N., Bhatia D.S. (1960). Assessment of the progressive spoilage of ice-stored shrimp. J. Fish. Res. Board Can..

[99] Puga Lopez D., Ponce Palafox J.T., Barba-Quintero G., Torres-Herrera M.R., Romero-Beltrán E., Arredondo Figueroa J.L., Garcia-Ulloa Gomez M. (2013). Physicochemical, proximate composition, microbiological and sensory analysis of farmed and wild harvested white shrimp *Litopenaeus vannamei* (Bonne, 1931) tissues. Curr. Res. J. Biol. Sci..

[100] Don S., Xavier K.M., Devi S.T., Nayak B.B., Kannuchamy N. (2019). Identification of potential spoilage bacteria in farmed shrimp (*Litopenaeus vannamei*): Application of Relative Rate. Int. J. Curr. Microbiol. Appl. Sci..

[101] Hoque M.S., Jacxsens L., Rahman M.B., Nowsad A.A.K.M., Azad S.M.O., De Meulenaer B., Rahman M. (2018). Evaluation of artificially contaminated fish with formaldehyde under laboratory conditions and exposure assessment in freshwater fish in Southern Bangladesh. Chemosphere.

[102] Immaculate J., Jamila P. (2018). Quality characteristics including formaldehyde content in selected Sea foods of Tuticorin, southeast coast of India. Int. Food Res. J..

[103] Nielsen M.K., Jørgensen B.M. (2004). Quantitative relationship between trimethylamine oxide aldolase activity and formaldehyde accumulation in white muscle from gadiform fish during frozen storage. J. Agric. Food Chem..

[104] del Mar Rey-Mansilla M., Sotelo C.G., Aubourg S.P., Rehbein H., Havemeister W., Jørgensen B., Nielsen M.K. (2001). Localization of formaldehyde production during frozen storage of European hake (*Merluccius merluccius*). Eur. Food Res. Technol..

[105] GOSL. The Gazette of the Democratic Socialist Republic of Sri Lanka, 2010, No 1649/19, 529 Food (Formaldehyde in Fish) Regulations. https://www.gazette.lk/.

